# Cognitive health promotion through a low-intensity high-volume webinar intervention for older adults at risk of future dementia

**DOI:** 10.3389/fpsyg.2025.1613890

**Published:** 2025-08-12

**Authors:** Keera N. Fishman, Christopher Pilieci, Linda Truong, Gillian Rowe, Renee Climans, Iris Yusupov Rose, Kelly J. Murphy

**Affiliations:** ^1^Neuropsychology and Cognitive Health, Baycrest Hospital, Toronto, ON, Canada; ^2^Ontario Shores Centre for Mental Health Sciences, Whitby, ON, Canada; ^3^Kunin-Lunenfeld Centre for Applied Research and Evaluation, Rotman Research Institute, Baycrest Academy for Research and Education, Toronto, ON, Canada; ^4^Department of Psychology, University of Toronto, Toronto, ON, Canada

**Keywords:** memory decline, cognitive decline, older adults, webinar intervention, aging and technology, psychoeducation, non-pharmacological interventions, neurodegenerative diseases

## Abstract

**Introduction:**

Mild cognitive impairment (MCI) affects 1 in 10 older adults and is a significant risk factor for dementia, a condition impacting over 63 million people worldwide. Despite the growing need for dementia prevention care, resources to empower individuals with MCI/cognitive decline and their families remain limited. The Learning the Ropes Foundations^©^ webinar was developed to provide a free, evidence-based, and accessible low-volume, high-intensity intervention to support brain health.

**Methods:**

Between January and December 2024, 78 participants with cognitive decline (99% >60 years, 58% women) and 30 family members of those with cognitive decline (97% >50 years, 57% women) completed a survey assessing the webinar’s usability, satisfaction, and ability to motivate behavior change. One-month following webinar completion, 19 participants with cognitive decline completed a follow-up survey assessing their implementation of behavior changes. Surveys included Likert-scale and open-ended questions. Data were analyzed using descriptive statistics.

**Results:**

Among survey respondents, 82% of participants with cognitive decline and 97% of family agreed they could apply the information to their everyday lives, 81% of participants with cognitive decline and 100% of family agreed they would recommend the webinar, and 90% of all participants reported being motivated to adopt at least one behavior change. Of the one-month follow-up participants, 74% reported implementing at least one behavior change.

**Discussion:**

The Learning the Ropes Foundations^©^ webinar shows strong potential as a user-friendly resource that supports usability, satisfaction, and motivation for behavior change among individuals with MCI/cognitive decline and their families. Future directions include expanding reach and evaluating long-term lifestyle impacts.

## 1 Introduction

### 1.1 Background

Lifestyle practices can significantly influence the risk of future dementia, with evidence suggesting that healthy lifestyles may prevent up to 45% of dementia cases worldwide (Calandri et al., 2024; Livingston et al., 2024). Despite this knowledge, more work is needed to translate dementia prevention information into practical strategies that can benefit at-risk older adults, particularly those who self-identify as experiencing cognitive decline or mild cognitive impairment (MCI). Estimates suggest that approximately 1 in 10 older adults currently experience MCI worldwide, highlighting a large (and growing) population who are in present need of dementia prevention care (Chen et al., 2023; Manly et al., 2022; Pessoa et al., 2019). Indeed, while MCI is an established high-risk factor for dementia (Öksüz et al., 2024), active intervention involving dementia prevention care may help to manage one’s risk of future progression (Livingston et al., 2024). The key difference between MCI and dementia is that individuals with MCI show greater than expected cognitive decline for their age but they can still manage their daily activities independently, whereas individuals with dementia experience both cognitive decline and a noticeable loss of independence in their everyday functioning (Pessoa et al., 2019).

### 1.2 Multicomponent interventions

Although cognitive decline is a shared risk factor for dementia across all cases of MCI, the presence or absence of co-occurring physical (e.g., hypertension, hyperlipidemia, diabetes, sensory loss) and mental (e.g., depression, anxiety) risk factors are more varied in this population. Thus, providing interventions that focus only on one risk factor, whether it be physical or mental, is insufficient given that not everyone with MCI will have the same health risks. For example, not everyone with MCI will have high blood pressure, not everyone will be sedentary, and not everyone will experience sensory loss. Similarly, providing interventions that focus only on the common risk factor of cognitive decline is also insufficient given the known impacts of physical and mental health risk factors on the progression of MCI to dementia (Ngandu et al., 2015; Öksüz et al., 2024; Williams et al., 2010). As a result, multicomponent interventions targeting several risk factors at once through healthy lifestyle changes may be more effective in helping to delay or prevent the onset of future dementia for this at-risk population (Levy et al., 2022). These interventions offer individuals the flexibility to tailor strategies to their personal risk profile while supporting them in identifying and overcoming barriers to accessing appropriate resources and services.

Multicomponent interventions targeting dementia risk factors are shown to have the greatest efficacy in terms of effecting broad-based positive impacts (e.g., improvements in functional memory, dietary changes, physical activity) for at-risk older adults (Castro et al., 2023; Gardener et al., 2024; Kivipelto et al., 2018). Currently, there are some evidence-based, manualized multicomponent group interventions targeting the management of MCI and prevention of dementia worldwide. These include the Learning the Ropes for Living with MCI^®^ (LTR) program in Canada (Murphy et al., 2014), the Healthy Action to Benefit Independence and Thinking (HABIT^®^) program in the United States (Levy et al., 2022), and the La Trobe and Caulfield Hospital (LaTCH) program in Australia (Kinsella et al., 2020). These programs provide psychoeducational tools and practical tips for participants with MCI and their families to adopt memory strategies and brain healthy lifestyles (reviewed in Anderson et al., 2024). Moreover, these multicomponent intervention programs have been shown to effect positive behavior changes among participants, including improvements in functional memory, memory strategy knowledge, memory strategy use, dietary habits, and physical activity (e.g., Fogarty et al., 2016; Levy et al., 2022; Ngandu et al., 2015; Troyer et al., 2008). There are also specific components of these interventions that have been found to target certain outcomes. For example, the HABIT^®^ program found relationships between participation in the wellness education and support group components with improved participant quality of life and mood, as well as participation in the memory strategy training component with improvements in memory-based instrumental activities of daily living (iADLs; Levy et al., 2022; Ngandu et al., 2015). Despite the overall benefits of these interventions, however, they have limitations that restrict their broad availability. These limitations may include the availability of trained professionals needed to facilitate these programs, the number of participants the programs can serve at once, the time commitment required, the requirement of a formal diagnosis to enrol, and the geographic location of the program (Levy et al., 2022; Murphy, 2018).

### 1.3 The current study

To address these limitations and expand the reach of dementia prevention care, the study authors developed the Learning the Ropes Foundations^©^ webinar. This webinar is an asynchronous psychoeducational intervention which was designed based on research attesting to the need, potential, and efficacy of low-intensity, high-volume interventions in meeting health care needs (Vincent et al., 2021). Many of these interventions have been successfully adopted in tertiary health clinics (e.g., anxiety, sleep), in primary care, as well as in provincial mental health crisis settings. They have also been found to be acceptable and well-received by at-risk older adults (Siette et al., 2024; Wuthrich et al., 2024). The Learning the Ropes Foundations^©^ webinar was created as a low-volume, high-intensity asynchronous intervention that is freely available, for anyone (e.g., regardless of MCI diagnosis), to access any time and anywhere, at: www.baycrest.org/ltrfoundations.

The Learning the Ropes Foundations^©^ webinar was guided by evidence-based research regarding some of the most critical aspects of MCI. Moreover, the webinar encompassed three key teachings from the Learning the Ropes for Living with MCI^®^ (LTR) program (Climans et al., 2014; Murphy, 2018; Murphy et al., 2014). These teachings include using a memory organizer, participating in recreation, and building adaptive coping strategies. Using a memory organizer was included in the webinar because memory change is the most common client-reported symptom of MCI (Ahmed et al., 2008; Buckley et al., 2015; Yates et al., 2017). Participating in recreation was included because one of the most common impacts of MCI is withdrawal from participation in leisure activities (Innes et al., 2016; Parikh et al., 2016). Last, building adaptive coping strategies (i.e., taking a deep breath and shifting from being reactive to responsive in a challenging situation) was included because experiencing cognitive decline can be worrisome and stressful for individuals with MCI as well as their family members (Frank et al., 2006; McIlvane et al., 2008; Murphy, 2015). Altogether, the Learning the Ropes Foundations^©^ webinar was designed as a brief, targeted, and readily available psychoeducational intervention to help individuals living with cognitive decline and their families to effect positive behavior changes and reduce their risk of progressing to dementia.

In this brief report, we present study outcomes related to the Learning the Ropes Foundations^©^ webinar’s usability, satisfaction, and ability to motivate behavior change with individuals living with cognitive decline and their family members. To do this, we surveyed participants who completed the webinar about any cognitive-related concerns and emotional difficulties they were experiencing prior to webinar completion, as well as the lifestyle behaviors they were implementing prior to webinar completion. These survey data were used to evaluate whether the target population’s concerns and reported behaviors were addressed by the content of the webinar. We then surveyed participants about their experience with the webinar and whether they were motivated to effect positive behavior changes in their lives related to the webinar’s key teachings. As our primary outcomes, we asked participants about the webinar’s usability, their satisfaction with it, and their motivation for behavior change immediately following webinar completion. As a secondary outcome, we also explored whether participants had implemented the key teachings in a one-month follow up survey.

## 2 Methods

### 2.1 Learning the Ropes Foundations^©^ History

The Learning the Ropes Foundations^©^ webinar was developed based on the Learning the Ropes for Living with MCI^®^ (LTR) program, a 7-session multicomponent intervention currently operating within Canada (Climans et al., 2014; Murphy, 2014, 2018; Murphy et al., 2014). This program provides evidence-based memory strategies and promotes brain healthy lifestyle behaviors (e.g., physical exercise, cognitive engagement, social interaction, leisure activities) for individuals living with MCI and their family members (Murphy et al., 2014). An evaluation of the LTR program showed that participants overall demonstrated increased memory strategy knowledge and memory strategy use compared to waitlist controls (Troyer et al., 2008). However, due to the same limitations as many of the multicomponent interventions previously mentioned (e.g., LaTCH, HABIT^®^), the program is currently not broadly available and the demand for it exceeds its capacity. As a result, three of the study authors (KM, GR, & RC) developed a condensed version of the LTR program that could be delivered to larger audiences (e.g., 40 to 50 individuals) which provided some of the key information from the full program while individuals were on the waitlist. This condensed version, known as the Learning the Ropes Foundations^©^, included a 90-minute in-person, interactive, facilitator-led discussion focusing on three key teachings from the full program. The Learning the Ropes Foundations^©^ was then further streamlined into an asynchronous webinar intended as a low-volume, high-intensity intervention to address the aforementioned gaps in accessibility.

### 2.2 Learning the Ropes Foundations^©^ Webinar

The Learning the Ropes Foundations^©^ webinar conveys three key teachings from the LTR program: using a memory organizer, participating in recreational activities, and building adaptive coping strategies. It was designed using the Articulate Storyline, an e-learning software.^1^ The webinar was developed so that participants could complete the program at their own pace and could review the material again as needed. The interface was programmed so that individuals with cognitive decline and their family members could type in a simple website link (no registration or login required) and be directed to the webinar intervention easily and immediately. Closed captions, transcripts, downloadable summaries, as well as translations into French and Spanish were integrated to optimize accessibility. Polling questions, feedback opportunities, as well as exercises and activities led by the asynchronous facilitator were integrated to include interactive components to the webinar. Ultimately, the wide availability of this asynchronous intervention means that a greater number of older adults experiencing cognitive decline and their family members can participate, regardless of the availability of program facilitators, the requirement of a formal diagnosis, the program’s geographic location, or access to specialized services.

The Learning the Ropes Foundations^©^ webinar launched in January 2024. The webinar was promoted through the websites of three organizations involved in cognitive aging and brain health, including Baycrest (a geriatric hospital in Toronto), BrainXchange™ (a knowledge exchange network focused on the quality of life of older adults living with neurological or mental health conditions), and Cogniciti (a brain health subsidiary company of Baycrest that provides a free brain health assessment online). Individuals who completed the webinar had the option of participating in the research study following their completion of the webinar. The research study component of the webinar was approved by Baycrest’s Research Ethics Board (REB #23-12). All individuals who opted to participate signed an electronic informed consent form prior to their participation. Participants were not compensated for their time. Data were downloaded for the present study in December 2024, approximately one-year following the initial webinar launch.

### 2.3 Participants

Between January 2024 and December 2024, approximately 1,700 unique individuals accessed the Learning the Ropes Foundations^©^ webinar. Immediately following webinar completion, all individuals were invited to complete a post-webinar survey about the primary study outcomes of usability, satisfaction, and motivation to implement positive behavior change based on the webinar’s key teachings (“immediate post-webinar survey”). The invitation to participate in the study was included on the final slide of the webinar. This served as our participant recruitment method. Once they consented to participate in the survey, participants reported demographic information (e.g., age, gender) and whether they identified as a person experiencing cognitive decline (Cognitive Decline group) or as a family member/friend of a person with cognitive decline (Family group). For those identifying with cognitive decline, participants were asked whether they were formally diagnosed with mild cognitive impairment (MCI), with dementia, or if they had not received a formal diagnosis. Upon survey completion, participants were invited to complete a one-month follow-up survey regarding their implementation of the webinar’s key teachings (“one-month follow-up survey”).

A total of 124 participants completed the immediate post-webinar survey, of which 108 were included in the present analysis (Cognitive Decline group: *n* = 78, Family group: *n* = 30). Sixteen participants were removed from the analysis because their reported identity did not match one of the study’s target groups (*n* = 12; e.g., a person interested in the subject matter but not experiencing cognitive decline) or because they included more than one identity (*n* = 4; e.g., a person with a mild traumatic brain injury and supporting someone with cognitive decline). Of the 78 participants identifying with cognitive decline, 50 indicated they were diagnosed with MCI, 1 indicated they were diagnosed with dementia, and 27 indicated they had not received any formal diagnosis. Further, of the 78 participants with cognitive decline, 19 completed the one-month follow-up survey. Unless referring to this follow-up group specifically, findings pertain to all 78 participants identifying with cognitive decline (Cognitive Decline group) and all 30 family members (Family group) who completed the immediate post-webinar survey.

As seen in Table 1, women comprised the majority of both the Cognitive Decline (58%) and Family (57%) groups. Both groups identified as majority White (Cognitive Decline = 74%, Family = 87%) with a smaller proportion identifying as Asian (Cognitive Decline = 9%, Family = 10%) or Black (Cognitive Decline = 3%, Family = 0%). Most participants held either a Bachelor’s degree (Cognitive Decline = 24%, Family = 37%) or a Master’s/Doctoral degree (Cognitive Decline = 15%, Family = 33%). Age distributions varied slightly such that those with cognitive decline were mostly above 60 (99%) and family members were mostly above 50 (97%). See Table 1 for a summary of all demographics reported.

**Table 1:** Participant demographics reported as the frequency (*n*) and proportion (%) of each group.

	Cognitive Decline group (*n* = 78)		Family group (*n* = 30)	
Total (*N* = 108)	*n*	%	*n*	%
Age				
Under 50 years	1	1.28	1	3.33
50–59 years	0	0.00	6	20.00
60–69 years	17	21.79	5	16.67
70–79 years	37	47.44	13	43.33
80 years and above	23	29.49	5	16.67
Gender				
Woman	45	57.69	17	56.67
Man	31	39.74	13	43.33
Other	2	2.56	0	0.00
Ethnicity				
White—North American, European	58	74.36	26	86.67
Asian—South, East, Southeast	7	8.97	3	10.00
Black—North American, Caribbean	2	2.56	0	0.00
Indian—Caribbean	1	1.28	0	0.00
Other	10	12.82	1	3.33
Education				
Some High school	2	2.56	0	0.00
High school graduate	6	7.69	1	3.33
Associate degree	8	10.26	5	16.67
Some college	8	10.26	3	10.00
Bachelor’s degree	25	32.05	8	26.67
Master’s/Doctoral degree	19	24.36	11	36.67
Professional degree	4	5.13	2	6.66
Other	6	7.69	0	0.00

Left: Cognitive Decline group (*n* = 78). Right: Family group (*n* = 30).

### 2.4 Measures and procedures

Participants who completed the immediate post-webinar survey were asked to report some of the cognitive-related concerns and emotional difficulties they were experiencing themselves or noticing in their family member prior to webinar completion. Participants were then asked about their implementation of the key teachings prior to their participation in the webinar. Multiple choice questions were presented such as “What are some of the main concerns you may be experiencing related to mild cognitive impairment?,” “What kinds of difficulties are you noticing in your/their thinking skills?,” and “Do you feel you have enough recreation in your life?”.

Following these questions, participants were asked to provide feedback regarding the primary outcomes of usability, satisfaction, and motivation for behavior change to implement the webinar’s key teachings immediately following webinar completion. These feedback questions were developed based on the D’Amico et al. (2022) usability and satisfaction survey related to a guided e-learning program for older adults. Similar questions regarding overall usability and satisfaction as well as satisfaction with specific webinar elements (i.e., content and delivery) were used given that a broadly comparable interface was used to design and implement the LTR-F webinar. Multiple choice questions included five-point Likert scales with participants rating their level of agreement (i.e., strongly agree to strongly disagree) with statements such as “The webinar shared information in a clear way” (usability), “I learned information I could apply in my everyday life,” and “I would recommend the webinar to a friend” (satisfaction). Participants also rated their level of motivation (i.e., very motivated to very unmotivated) for statements such as “How motivated are you to use a memory organizer?” and “How motivated are you to engage in recreational activities?” (motivation for behavior change). Finally, open-ended questions were presented for internal program evaluation in which participants responded to statements such as “What is the best thing about the Learning the Ropes Foundations^©^ webinar?” and “What would make this webinar more useful in your daily life?”.

Participants who completed the one-month follow up survey were asked to provide feedback about their behavior change in terms of implementing the key teachings since webinar completion (exploratory outcome). Participants in this survey included only a small sample of those with cognitive decline. Similar five-point Likert scale questions were included with participants rating their level of agreement (i.e., strongly agree to strongly disagree) with statements such as “As a result of my participation in the webinar, I am using a memory organizer” and “As a result of my participation in the webinar, I am making more time to engage in recreational activities”.

### 2.5 Data analysis

Data analyses included descriptive summaries of the quantitative data (multiple choice questions) and summaries of the responses to open-ended questions. The data were analyzed separately for each group, including those identifying with cognitive decline (Cognitive Decline group) and those identifying as a family member/friend (Family group). Quantitative data were analyzed for all outcomes as the frequency or proportion of each group. Open-ended responses were summarized for the purpose of internal program evaluation and are only included to supplement the quantitative findings herein. The data were summarized separately for each survey.

## 3 Results

### 3.1 Participant-reported emotional difficulties and cognitive-related concerns

Both groups reported similar trends in terms of the emotional difficulties and cognitive-related concerns they were experiencing themselves or noticing in their family members. For example, when asked about the emotional difficulties those with cognitive decline or their family members were experiencing, both groups reported being frustrated with the cognitive decline (Cognitive Decline = 72%, Family = 60%), being worried about the future (Cognitive Decline = 64%, Family = 53%), recognizing a sense of loss (Cognitive Decline = 54%, Family = 37%), and feeling depressed, anxious, or sad (Cognitive Decline = 51%, Family = 50%). When asked about some of the cognitive-related concerns those with cognitive decline were experiencing or their family members were noticing in the person with cognitive decline, both groups reported forgetting the names of people they just met (Cognitive Decline = 79%, Family = 53%), forgetting specific words they want to use in conversation (Cognitive Decline = 67%, Family = 40%), forgetting the reason they went into a room (Cognitive Decline = 67%, Family = 40%), and forgetting the details of something they just read (Cognitive Decline = 64%, Family = 50%). See Table 2 for a summary of all cognitive-related concerns reported.

**Table 2:** Cognitive-related concerns reported as the frequency (*n*) and proportion (%) of each group.

	Cognitive Decline group (*n* = 78)		Family group (*n* = 30)	
Total (*N* = 108)	*n*	%	*n*	%
Cognitive-related concerns				
Forgetting the names of people I/they just met	62	79.49	16	53.33
In conversation, coming up with a specific word I/they want to use	52	66.67	12	40.00
Going into a room and forgetting what I/they went there for	52	66.67	12	40.00
Forgetting details of something I/they have just read	50	64.10	15	50.00
Putting things down and then forgetting where I/they put them later	46	58.97	16	53.33
Forgetting the names of people I/they should know	45	57.69	13	43.33
Forgetting where I/they put things, such as glasses, keys, phone, or other important items	44	56.41	19	63.33
Forgetting details of recent conversations	43	55.13	22	73.33
Forgetting whether or not I/they have done something, such as turning off the light	43	55.13	11	36.67
Trouble remembering the details of past events, like a visit with friends	34	43.59	15	50.00
Retelling things to people because I/they forgot that it was already told	29	37.18	19	63.33
Forgetting the names of everyday items	24	30.77	4	13.33
In conversation, forgetting what I/they want to say	27	34.62	5	16.67
Leaving items behind that I/they meant to bring, such as wallets	18	23.08	9	30.00
Other	10	12.82	0	0.00
I have not noticed any difficulty with my/their thinking skills	5	6.41	0	0.00

Left: Cognitive Decline group (*n* = 78). Right: Family group (*n* = 30).

### 3.2 Key teachings used prior to webinar participation

Participants with cognitive decline generally reported low rates of implementing the webinar’s key teachings prior to webinar completion. For example, when asked whether they would be able to record the date of a plan in their schedule after running into a friend, only 49% reported they would be able to do so. When asked about whether they had enough recreation in their lives, only 38% reported that they did. Considering the webinar’s key teachings were to use a memory organizer, engage in recreational activities, and build adaptive coping strategies, this was an ideal sample to motivate for behavior change in this regard.

### 3.3 Primary outcomes measuring usability, satisfaction, and motivation for change

**Figure 1:**
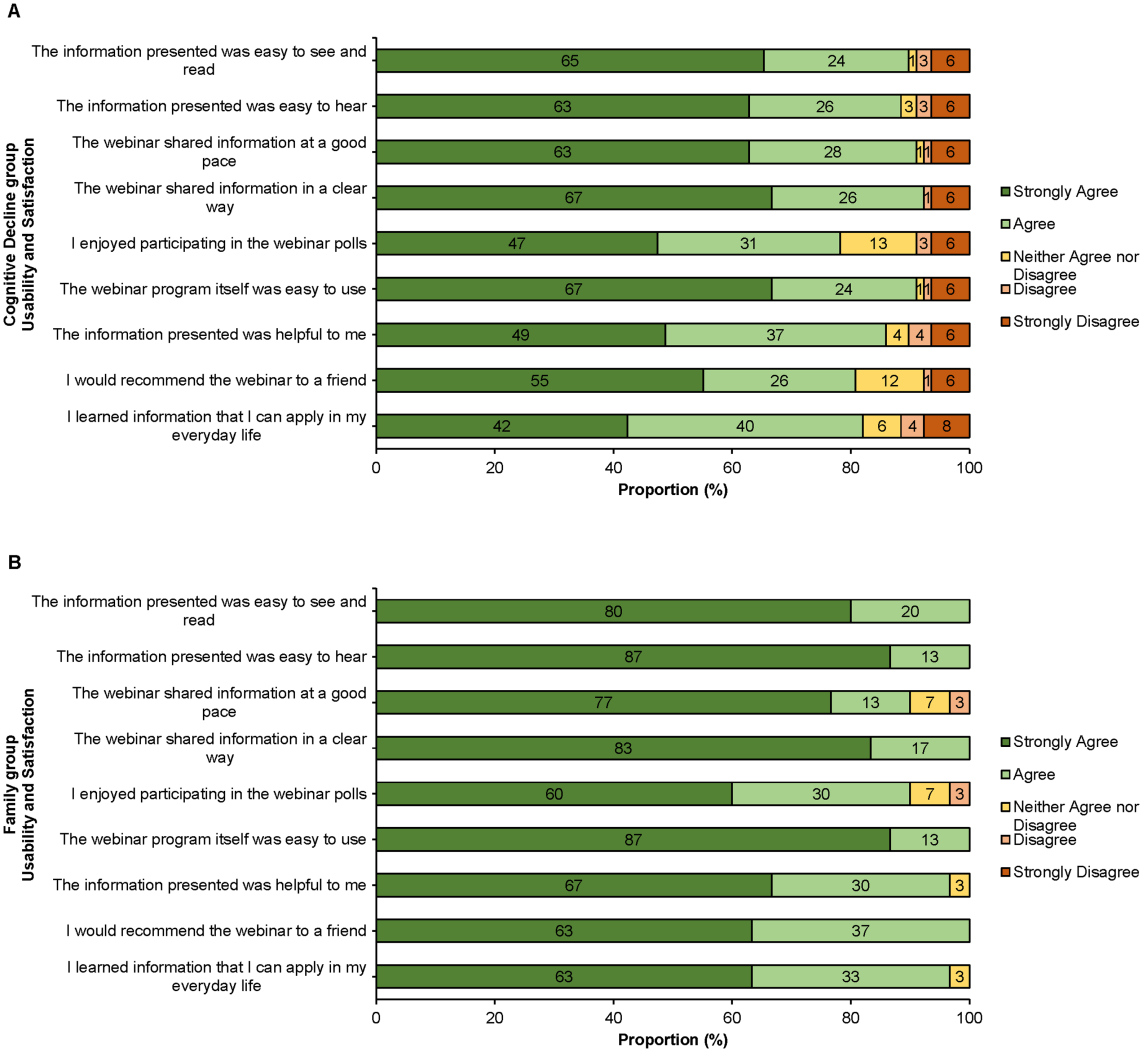
Usability and Satisfaction reported as a proportion (%) of each group. **(A)** Cognitive Decline group (*n* = 78). **(B)** Family group (*n* = 30).

Participants in both groups reported positive feedback about the webinar’s overall usability (see Figure 1). For example, when asked about the webinar’s format, 90% of those with cognitive decline and 100% of family members agreed that the information presented was easy to see and read, 88% of those with cognitive decline and 100% of family members agreed it was easy to hear, 91% of those with cognitive decline and 90% of family members agreed it was shared at a good pace, and 91% of those with cognitive decline and 100% of family members agreed the interface was easy to use.

Participants in both groups also reported positive feedback about their overall satisfaction with the webinar (see Figure 1). For example, when asked about the webinar’s content, 86% of those with cognitive decline and 97% of family members agreed that the information presented was helpful to them, 82% of those with cognitive decline and 97% of family members agreed they learned something they could apply in their everyday lives, and 81% of those with cognitive decline and 100% of family members agreed they would recommend the webinar to a friend.

**Figure 2:**
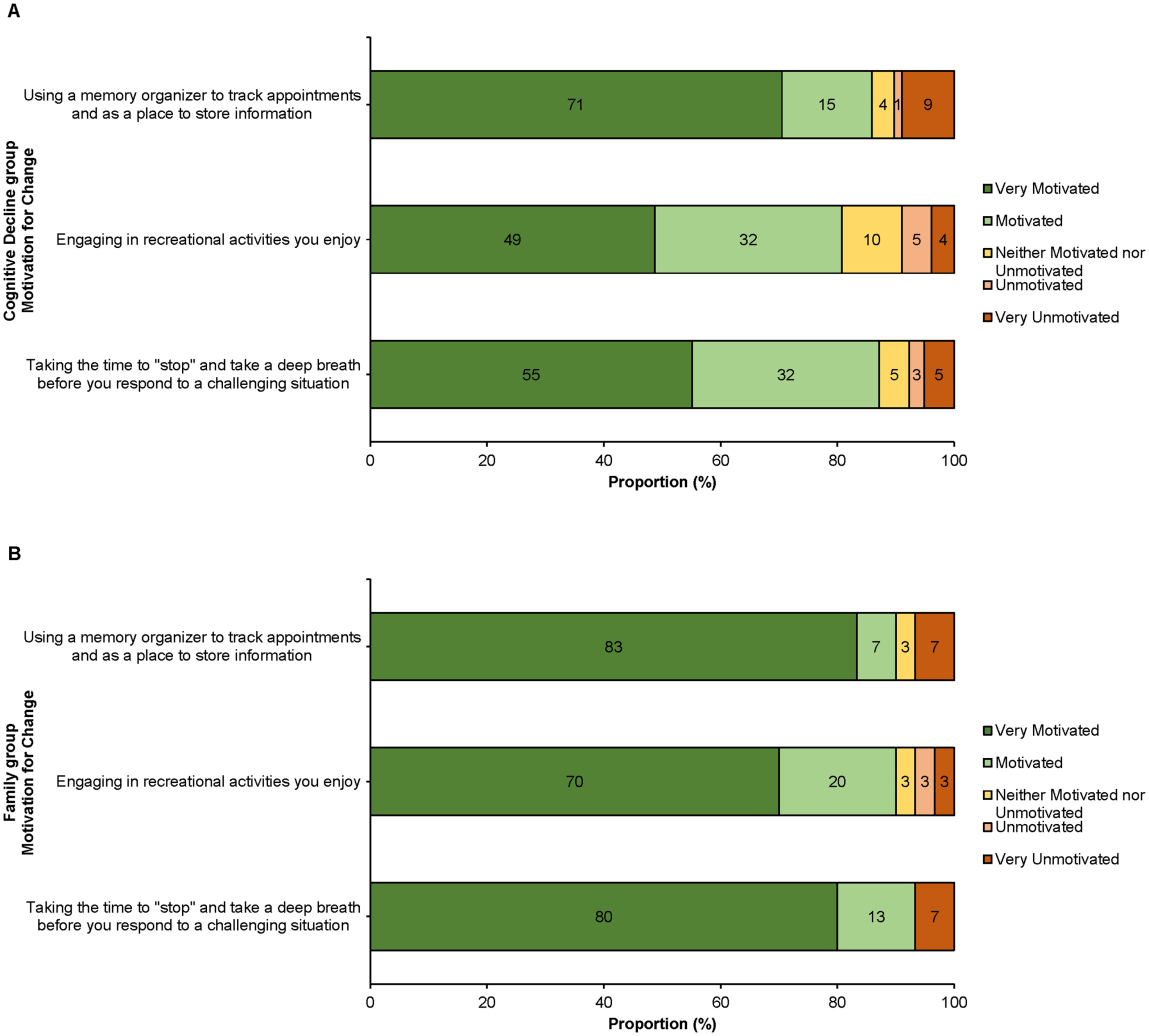
Motivation for behavior change reported as a proportion (%) of each group. **(A)** Cognitive decline group (*n* = 78). **(B)** Family group (*n* = 30).

Finally, participants in both groups reported positive feedback about their motivation for behavior change immediately following webinar completion (see Figure 2). When asked about whether they intended to implement some of the webinar’s key teachings, 86% of those with cognitive decline and 90% of family members were motivated to use a memory organizer, 81% of those with cognitive decline and 90% of family members were motivated to engage in recreational activities, and 87% of those with cognitive decline and 93% of family members were motivated to build adaptive coping strategies. Overall, 90% of respondents reported being motivated to implement at least one behavior change. Participants provided comments such as “…knowing that someone cares about MCI and has solutions” and “…it gave me tips to help me hold on to my brain power” which reinforced the webinar’s positive impact and value.

### 3.4 Key teachings used following webinar participation in the follow-up survey

One-month following webinar completion, participants with cognitive decline (*n* = 19) reported higher rates of implementing the webinar’s key teachings compared to pre-webinar rates (as an average across participants). When asked about whether they implemented the key teachings as a result of the webinar, 63% reported they were now using a memory organizer, 47% reported they were engaging in recreational activities, and 37% reported they were building adaptive coping strategies. Overall, 74% of follow-up participants reported implementing at least one behavior change. Given that prior to the webinar, only 49% of those with cognitive decline reported that they would be able to record the date of a plan in their schedule and only 38% reported they were getting enough recreation in their lives, these findings suggest that this low-intensity, high-volume intervention may be able to effect positive behavior change in participants as intended, though notably in a very small sample.

## 4 Discussion

In this brief report, we present outcomes related to the Learning the Ropes Foundations^©^ webinar’s usability, satisfaction, and ability to motivate behavior change. This low-intensity, high-volume webinar was designed to empower individuals experiencing cognitive decline and their families with clear, actionable strategies to reduce their risk of future dementia. To evaluate our primary outcomes, we asked participants about the webinar’s usability, their satisfaction with it, and their motivation to implement the webinar’s key teachings immediately following webinar completion. The majority of participants found the webinar usable, were satisfied with their experience, and were motivated to implement the webinar’s key teachings. To evaluate our secondary outcome, a small sample of participants with cognitive decline were asked about their implementation of the webinar’s key teachings one-month following the webinar. The majority of follow-up participants reported they were now using a memory organizer, approximately half reported they were engaging in recreational activities, and about one third reported they were using adaptive coping strategies. These findings suggest, though notably in a small sample, that the webinar may have effected positive behavior change among participants as intended.

Overall, the webinar successfully provided clear, applicable information that was well-received by our target population. Given that approximately 63 million older adults globally are in current need of dementia prevention care, this webinar may offer a more readily available solution for individuals to access without being limited by formal diagnosis, the availability of program facilitators, the intensity of the program, or the geographic location of the program.

### 4.1 Interpretation of primary outcomes: Usability, satisfaction, and motivation for behavior change

The overwhelmingly positive reception of the webinar underscores its usability, satisfaction, and ability to motivate positive behavior change. With respect to usability, the webinar was designed to maximize accessibility and ease of use, allowing participants to engage at their own pace and with immediate access through a simple website link. The webinar was also designed to communicate complex health concepts in a clear, relatable, and engaging manner. Accessibility features such as transcripts and closed captions further supported diverse user needs and reduced barriers to engagement. Survey results further reinforce these strengths, with approximately 95% of participants finding the webinar easy to navigate and to understand, demonstrating its user-friendliness as an online health intervention. These results complement previous findings suggesting that older adults are generally supportive of online health interventions when they are perceived as being easy to use, accessible, and useful (Bujnowska-Fedak and Pirogowicz, 2014). Moreover, these results complement previous research that older adults with subjective memory complaints and MCI are receptive to online health resources, as long as these tools are designed with simplicity and clarity in mind (LaMonica et al., 2017).

With respect to satisfaction, survey results indicate that approximately 90% of all participants found the content helpful, emphasizing its relevance and practical value. User testimonials such as “…knowing that someone cares about MCI and has solutions” and “…it gave me tips to help me hold on to my brain power” further reinforce the webinar’s positive impact and participant appreciation for the intervention. These sentiments support previous findings that individuals living with cognitive decline value clear, empathic communication and practical strategies that resonate with their lived experience (Mishari and Ali, 2025).

Beyond usability and satisfaction, results indicate that the webinar was able to encourage positive behavior change, with 90% of participants reporting motivation to adopt at least one new strategy, whether it be using a memory organizer, engaging in recreational activities, or building adaptive coping strategies. Notably, the webinar’s free and private format enables individuals who may be reluctant to seek public resources to access valuable cognitive health information in a comfortable setting, potentially encouraging them to take proactive steps toward supporting their cognitive health and well-being. Moreover, normalizing the MCI experience, currently affecting approximately 1 in 10 older adults worldwide, was a central theme of the webinar, and this framing may have contributed to increased receptivity and motivation to effect behavior changes (Chen et al., 2023; Manly et al., 2022; Pessoa et al., 2019; Warren, 2023).

### 4.2 Interpretation of results: Emotional difficulties

The survey results also show that individuals with cognitive decline and their family members endorsed similar trends regarding emotional difficulties they were experiencing. These included feelings of frustration with cognitive decline, worry about the future, recognition of a sense of loss, and experiencing symptoms of depression, anxiety, and/or sadness. These shared concerns highlight the emotional burden that cognitive decline can have not only on the individuals directly affected but also on their families, friends, and care partners. As well, these sentiments are consistent with prior research showing that both individuals with MCI and their family members often experience emotional challenges such as anxiety and feelings of a sense of loss, which can impact their well-being (Frank et al., 2006; McIlvane et al., 2008; Murphy, 2015).

### 4.3 Implications for clinical practice and public health

Given its wide accessibility and cost-effectiveness, the LTR-F webinar represents a sustainable solution for addressing cognitive health concerns in aging populations. Traditional in-person or live virtual workshops on cognitive health often require substantial time and financial and/or administrative resources, and often limit access for individuals living in remote or underserved communities. In contrast, the asynchronous, freely available nature of this webinar has facilitated broader dissemination with over 2,800 unique views to date. This intervention has been accessed by individuals residing nationally in Canada (including remote communities such as in Trois-Rivières and Port-Cartier, as well as urban areas including Toronto, Victoria, Calgary, London, Montreal, Winnipeg, and Charlottetown), and internationally including in the United States, Guatemala, Spain, Germany, Kuala Lumpur, Austria, and Thailand. Moreover, the webinar’s translations into French and Spanish expand its inclusivity, ensuring that non-English-speaking populations can also benefit from this low-intensity, high-volume intervention.

The webinar’s reach extends beyond participants living with cognitive decline, as it also provides family members with knowledge and ideas for how to support loved ones with cognitive decline. By equipping families with evidence-based strategies, the webinar may help to reduce care partner burden and improve care outcomes (Frank et al., 2006; McIlvane et al., 2008). Furthermore, normalizing the experience of MCI and emphasizing actionable strategies may help to mitigate stigma and encourage proactive health behaviors (Warren, 2023). By empowering individuals to manage their cognitive health independently, the webinar has the potential to reduce (or delay) some reliance on formal healthcare services, which may ultimately contribute to more efficient resource utilization. Additionally, delaying cognitive decline progression through the use of evidence-based strategies may lead to long-term reductions in healthcare costs associated with dementia care (Livingston et al., 2024).

### 4.4 Limitations and future directions

Despite its successes, this study has several limitations. First, there may have been a selection bias of individuals who chose to participate in the study, wherein individuals who were already motivated to engage in cognitive health interventions were more likely to participate. Also, while survey responses provided valuable insights, they relied on self-reported data, which may be subject to recall bias or social desirability effects (Althubaiti, 2016). This may be especially relevant for the follow-up survey exploring the secondary outcome, which garnered only a small sample of respondents. Moreover, participation required web access (e.g., via home equipment or a library) and digital literacy, which may have excluded individuals from lower socioeconomic backgrounds who are at a greater risk for cognitive decline (Wang et al., 2023). In addition, though the usability and satisfaction questionnaire was adapted from a comparable program for older adults reported in D’Amico et al. (2022), it was not developed based off of a standardized webinar usability/satisfaction questionnaire. Future studies could incorporate objective virtual cognitive assessments (e.g., Cogniciti’s Brain Health Assessment), incorporate additional standardized mood questionnaires [e.g., Geriatric Depression Scale-15 (Van Marwijk et al., 1995)] to investigate the relationship between motivation for behavior change and mood, or implement longitudinal follow-up studies to evaluate the sustained impact of the webinar on cognitive health outcomes as well as its ability to effect behavior change.

Future efforts will focus on expanding the webinar’s reach and evaluating the long-term impacts of the intervention. Planned next steps include translating feedback surveys into French and Spanish to ensure that responses from non-English-speaking participants are captured and analyzed. Additionally, broadening partnerships with other organizations will help to enhance dissemination efforts and foster cross-sector collaboration. Last, continued promotion through healthcare networks, community organizations, and primary care settings will also further optimize the webinar’s accessibility and engagement.

## 5 Conclusion

The Learning the Ropes Foundations^©^ webinar represents a cost-effective and accessible solution for older adults at risk of dementia and their family members. This low-intensity, high-volume intervention has demonstrated high usability, satisfaction, and the ability to motivate behavior change by communicating clear, actionable strategies to support cognitive health. The webinars are available in English^2^, French^3^, and Spanish^4^. Future directions include expanding the webinar’s reach and evaluating the potential for long-term health and lifestyle impacts.

## Data Availability

The datasets presented in this article are not readily available because data access was not granted for other researchers by Baycrest’s Research Ethics Board for the current study. Requests to access the datasets should be directed to Dr. Keera Fishman, kfishman@baycrest.org.

## Footnotes

^1^ https://www.articulate.com/360/storyline/

^2^ www.baycrest.org/ltrfoundations

^3^ www.baycrest.org/ltrfoundationsfr

^4^ www.baycrest.org/ltrfoundationsesp
